# TGF‐β‐mediated exosomal lnc‐MMP2‐2 regulates migration and invasion of lung cancer cells to the vasculature by promoting MMP2 expression

**DOI:** 10.1002/cam4.1758

**Published:** 2018-09-06

**Authors:** Dong‐ming Wu, Shi‐hua Deng, Teng Liu, Rong Han, Ting Zhang, Ying Xu

**Affiliations:** ^1^ Clinical Laboratory The First Affiliated Hospital of Chengdu Medical College Chengdu China

**Keywords:** exosomes, lncRNA, lung cancer, matrix metalloproteinase, TGF‐β

## Abstract

Previous studies indicated that transforming growth factor (TGF)‐β‐mediated exosomal microRNAs (miRNAs) regulate the migration and invasion of lung cancer cells; however, whether and how TGF‐β‐mediated exosomal long noncoding (lnc) RNAs regulate migration and invasion of lung cancer cells remains unclear. Here, coculture experiments showed that TGF‐β pretreatment increased the migration and invasion potential of lung cancer cells and TGF‐β pretreated A549 cells increases vascular permeability. Furthermore, we found that TGF‐β‐mediated exosomes, as carriers of intercellular communication, regulated lung cancer invasion, and vascular permeability. Transcriptional analysis also revealed that lnc‐MMP2‐2 was highly enriched in TGF‐β‐mediated exosomes and might function by increasing the expression of matrix metalloproteinase (MMP)2 through its enhancer activity, with ectopic expression and silencing of lnc‐MMP2‐2 affecting lung cancer invasion and vascular permeability. Additionally, lnc‐MMP2‐2 and MMP2 expression was assessed semiquantitatively, and tissue‐specific correlations between lnc‐MMP2‐2 and MMP2 expression were evaluated. These results suggested that exosomal lnc‐MMP2‐2 might regulate the migration and invasion of lung cancer cells into the vasculature by promoting MMP2 expression, suggesting this lncRNA as a novel therapeutic target and predictive marker of tumor metastasis in lung cancer.

## 1 INTRODUCTION

Lung cancer accounts for 13% of the incidence and 19.4% of the mortality associated with all malignant tumors.^1^ The population of cancer patients continues to rise, affected by the overall age of China's population and increased smoking incidence by young people.^1^, ^2^ Despite continuous improvements in the treatment of lung cancer, tumor metastasis remains the primary cause of poor prognosis. As a multi‐process life activity, lung cancer metastasis is affected and regulated by multiple factors; however, the mechanisms associated with how metastatic tumor cells cross the vascular endothelial cell layer into the blood‐circulatory system remain incompletely understood.^3^

During early tumor metastasis, tumor‐cell‐driver epithelial‐to‐mesenchymal transition (EMT) characterized by the loss of epithelial‐cells characteristic enables acquisition of characteristics of mesenchymal cells and is accompanied by the ability to migrate across the base and vascular endothelial cell layer, thereby promoting tumor metastasis.^4^, ^5^ Transforming growth factor (TGF)‐β is among the most important EMT inducers,^6^ as it interacts with corresponding membrane receptors, which activate downstream proteins associated with various EMT‐related signaling pathways and further promote the metastatic potential of tumor cells.^7^, ^8^

Exosomes are small membranous vesicles (30‐100 nm) present in all biological fluids.^9^, ^10^ Exosomes carry proteins and genetic materials, including DNA, mRNA and noncoding RNA, capable of revealing genetic information related to their parent cells.^11^ A recent study reported that noncoding RNA is enriched in secreted exosomes to protect RNAs from degradation,^12^, ^13^, ^14^ with another study showing that exosomes carry proteins that act as regulators of multiple pathways.^15^

Noncoding RNAs encoded by the eukaryotic genome comprise a large number of RNAs that are not translated into proteins. Accumulating evidence revealed major transcriptional and posttranscriptional regulatory roles of long noncoding (lnc)RNAs 16 and include transcription‐factor recruitment, chromatin remodeling, histone modification, pre‐mRNA splicing, and as molecular sponges and scaffolds involved in the development of normal tissues or organs, as well as in carcinogenesis and the aggression of diverse malignancies.^17^, ^18^

A previous study reported that TGF‐β influences microRNA (miRNA) expression in exosomes^19^; however, the role of TGF‐β in promoting exosomal lncRNA expression has not been reported. Here, we performed coculture of TGF‐β‐treated A549 cells with lung cancer cells or lung microvascular endothelial cells to evaluate the TGF‐β‐mediated invasion ability of the lung cancer cells and changes in permeability of the vascular endothelial cell layer. Additionally, we screened differential exosomal lncRNA expression based on lncRNA arrays to determine those affected by TGF‐β treatment, revealing that lnc‐matrix metalloproteinase (MMP)2‐2 was significantly enriched in exosomes following TGF‐β treatment. Furthermore, bioinformatics analysis indicated that lnc‐MMP2‐2 increases MMP2 expression, with these results indicating that TGF‐β mediated exosomal lnc‐MMP2‐2 might regulate lung cancer‐cell migration and invasion by promoting MMP2 expression and suggesting lnc‐MMP2‐2 as a potential diagnostic marker of lung cancer metastatic potential.

## 2 MATERIALS AND METHODS

### 2.1 Reagent

All the primary and secondary antibodies were obtained from Proteintech Group, Inc. (Wuhan, China). Rhodamine B isothiocyanate‐Dextran were purchased from Sigma‐Aldrich (St. Louis, MO, USA). All other kits and reagents were purchased from the Beyotime Institute of Biotechnology (Shanghai, China). Tissue arrays were from Outdo Biotech Co., Ltd. (Shanghai, China).

### 2.2 Cell culture and coculture

Non‐small cell lung cancer cells (A549) and human lung microvascular endothelial cells (HMVEC‐L) were maintained in Roswell Park Memorial Institute (RPMI)‐1640 medium supplemented with 10% (v/v) fetal bovine serum, 10 mmol/L l‐glutamine, and 5 mg/mL penicillin/streptomycin at 37°C with 5% CO_2_. All media and supplements were purchased from Invitrogen (Carlsbad, CA, USA). For coculture, a 24‐well plate with a transwell inserts (0.4‐μm pore size; Corning) was used, with 20 000 A549 or HMVEC‐L cells plated in the lower wells of the transwell chamber while 1 × 10^4^ A549 cells (control) or A549 cells pretreated with TGF‐β (24 hours, 10 ng/mL) in 200 μL media were added to the upper wells. The chamber was incubated for 24 hours at 37°C, and cells in the lower wells were harvested for subsequent experiments.

### 2.3 Exosome isolation and characterization

Exosomes were isolated according to a previous method.^20^ Exosomes from A549 cells pretreated for 24 hours with serum‐free media or serum‐free media containing 10 ng/mL TGF‐β were designated as “exo” and “Texo,” respectively. The morphology and particle size of exosomes dissolved in phosphate‐buffered saline were characterized via transmission electron microscopy (TEM; FEIG2; FEI, Hillsboro, OR, USA) as previously described.^21^

### 2.4 LncRNA microarray analysis

The lncRNA microarray analysis was performed by Aksomics Inc (Shanghai,China) using Human LncPathTM EMT Pathway LncRNA Microarray (8 × 15K, Arraystar). Briefly, total RNAs were extracted with TRIzol reagent from exosomes extracted from TGF‐β pretreated or untreated A549 cell culture supernatant according to the manufacturer's instruction. RNA quantity and quality were measured by NanoDrop ND‐1000. RNA integrity was assessed by standard denaturing agarose gel electrophoresis. Sample labeling and array hybridization were performed according to the Agilent One‐Color Microarray‐Based Gene Expression Analysis protocol (Agilent Technology) with minor modifications. Briefly, mRNA was purified from total RNA after removal of rRNA (mRNA‐ONLY Eukaryotic mRNA Isolation Kit, Epicentre, Madison, Wisconsin, USA). Then, each sample was amplified and transcribed into fluorescent cRNA along the entire length of the transcripts without 3′bias utilizing a random priming method (Arraystar Flash RNA Labeling Kit, Arraystar, Rockville, Maryland, USA). The labeled cRNAs were purified by RNeasy Mini Kit (Qiagen, Shanghai, China). The concentration and specific activity of the labeled cRNAs (pmol Cy3/μg cRNA) were measured by NanoDrop ND‐1000. 1 μg of each labeled cRNA was fragmented by adding 5 μL 10× Blocking Agent and 1 μL of 25× Fragmentation Buffer, then heated the mixture at 60°C for 30 minutes, finally, 25 μL 2× GE Hybridization buffer was added to dilute the labeled cRNA. 50 μL of hybridization solution was dispensed into the gasket slide and assembled to the LncRNA expression microarray slide. The slides were incubated for 17 hours at 65°C in an Agilent Hybridization Oven. The hybridized arrays were washed, fixed, and scanned using the Agilent DNA Microarray Scanner (part number G2505C).

### 2.5 Lnc‐MMP2‐2 overexpression and silencing

A549 and HMVEC‐L cells were transfected with a pCDNA3.1‐lnc‐MMP2‐2 plasmid (Shanghai Integrated Biotech Solutions Co., Ltd., Shanghai, China) or silenced with lnc‐MMP2‐2 Smart Silencer (RiboBio Co., Ltd., Guangzhou, China) according to manufacturer instructions and using the following target sequences: ATGTGGCTGAGCAGGGTCTG, CCTTCACACGACCTCCTG, TGCAAGAAACATCTCTT, AGTTCTCCATCCTGCTGCTCA, and GTGAGCTCCAGGGGTCTAGG. The corresponding negative control was purchased from RiboBio Co., Ltd.

### 2.6 Total RNA extraction and quantitative reverse transcription polymerase chain reaction (qRT‐PCR)

Total RNA was extracted using a total RNA extraction kit (Solarbio, Beijing, China) according to manufacturer instructions. RNA samples were then reverse transcribed using an iScript cDNA synthesis kit (Bio‐Rad, Hercules, CA, USA) and amplified by qRT‐PCR on a CFX96 real‐time system (Bio‐Rad) using SYBR Green Supermix (Bio‐Rad) and the following primers: lnc‐MMP2‐2 forward, 5′‐CTCGTCCCAGACCCTAGGTCTCCC‐3′ and reverse, 5′‐CTCGTCCCAGACCCTAGGTCTCCC‐3′; and β‐actin forward, 5′‐CCTGGCA CCCAGCACAAT‐3′ and reverse, 5′‐GGGCCGGACTCGTCATAC‐3′.

### 2.7 Immunofluorescence and Western blot

Immunofluorescence and Western blot arrays were performed to measure the expression of E‐cadherin, vimentin, N‐cadherin, occludin zonula occludens‐1 (ZO‐1), CD63,CD9,Alix and MMP2 according to our previous method.^22^

### 2.8 Wound healing assay

Cells were seed in 6‐well plates and cultured in the RPMI 1640 containing 10% serum, for 24 hours until 90% confluence. The cells were then wounded with 200 μL pipette tips, and the cell Debris was washed away with PBS. The wound scars were photographed with an inverted light microscope at 0 and 24 hours after the scratch was made. The ratio of the healing area relative to the initial wound area was calculated, and the wound area was quantified using ImagePro Plus 7.0 software(Media Cybernetics, Rockville, Maryland, USA). Quantification of bands was performed using the ImageJ program (National Institutes of Health, Bethesda, Maryland, USA).

### 2.9 Transwell assays

Cells were cultured in 10‐cm plates, with fresh medium added 18 hours before each assay. Cells were then trypsinized, washed twice, resuspended in serum‐free medium. The lower wells of a transwell chamber were filled with RPMI‐1640 containing 10% serum while 1 × 10^4^ cells in 200 μL serum‐free media were added to the upper wells. The chamber was incubated for 24 hours at 37°C, during which time cells on the upper membrane surface were scraped off to leave only those that had migrated through the membrane. The membrane was then fixed in methanol, stained with 0.1% crystal violet, and air‐dried. Stained cells were quantified and averaged over five fields from triplicate wells of each test condition.

### 2.10 HMVEC‐L cell permeability assay

HMVEC‐L cells were seeded onto Costar transwell inserts (0.4‐μm pore size; Corning). The following day, rhodamine B isothiocyanate‐dextran (400 μg/mL) was added to the upper wells. After 2 hours of additional incubation at 37°C, the medium in the lower wells was collected, and the fluorescence intensity was measured with 485 and 535 nm as the excitation and emission wavelengths, respectively, using a FlexStation 3 microplate reader (Molecular Devices, Sunnyvale, CA, USA).

### 2.11 Luciferase assay

The MMP2 promoter that range from 2 kb upstream to 200 bp downstream of the human MMP2 gene transcription start site were cloned into the pGL3‐Basic vector. A549 cells were cotransfected with a MMP2‐promoter containing luciferase promoter construct and either an empty control vector (PCDNA.3.1) or lncMMP2‐2 expression Plasmid. Luciferase activity was determined using a Luciferase assay kit (Promega, Madison, Wisconsin, USA), normalizing to protein concentration and then to a control sample transfected with pGL3.

### 2.12 Immunohistochemistry and in situ hybridization

Lung cancer tissue arrays (HLug‐Ade050CD‐01) were purchased from Shanghai Outdo Biotech Co., Ltd. (Shanghai, China), and an in situ hybridization kit was purchased from Shanghai Gefan Biotech Co., Ltd. (Shanghai, China). Immunohistochemistry was performed as previously described,^22^ and in situ hybridization was performed according to manufacturer instructions. The probe used for lncRNA‐MMP2‐2 was 5′‐FAM‐ accctaggctgcaggctcctgctttgggct‐3′.

### 2.13 Statistical analysis

Each experiment was performed at least three times independently, and results are represented as the mean ± standard error of the mean. Comparisons between two groups were performed using Student's *t* test, and differences were considered significant at *P* < 0.05. All statistical analyses were performed using GraphPad Prism software (GraphPad Software, San Diego, CA, USA).

## 3 RESULTS

### 3.1 TGF‐β pretreated A549 cells increases the migratory and invasive activity of lung cancer cells

**Figure 1:**
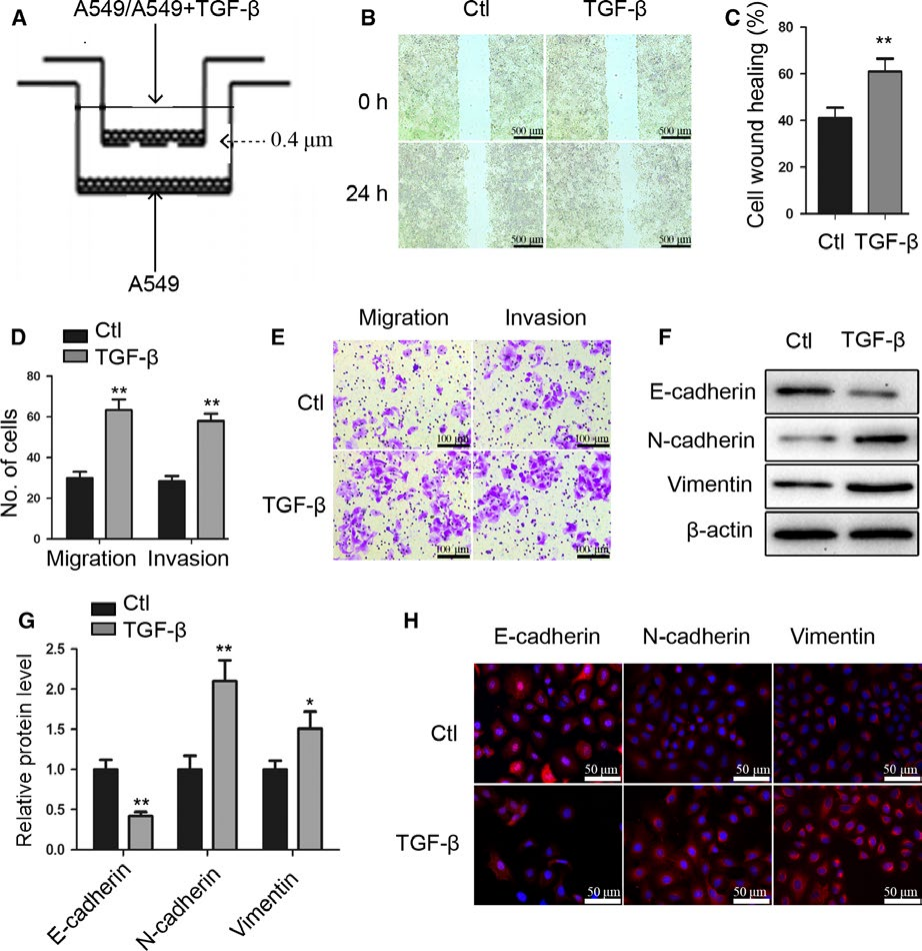
TGF‐β pretreated A549 cells increase the migratory and invasive activity of lung cancer cells. A, Schematic representation of the coculture assay. B, Wound healing results for Ctl/A549 (Ctl) and TGF‐β/A549 (TGF‐β) cells. Scale bar, 500 μm. C, Quantification of wound healing. D, Quantification of transwell migration and matrigel invasion. E, Transwell migration and matrigel invasion by Ctl and TGF‐β A549 cells. Scale bar, 100 μm. F‐H, Expression of E‐cadherin, N‐cadherin, and vimentin in Ctl and TGF‐β A549 cells as measured by (F‐G) western blot and (H)immunofluorescence staining (scale bar, 50 μm). *P < 0.05; **P < 0.01 versus Ctl

We cocultured A549 cells with TGF‐β‐pretreated A549 cells (TGF‐β+A549/A549, TGF‐β/A549) or untreated A549 cells (A549/A549,Ctl/A549) (Figure 1A), and wound healing (Figure 1B,C), transwell migration, and matrigel invasion assays (Figure 1D,E) revealed increased migration and invasiveness of TGF‐β/A549 cells relative to Ctl/A549. Because EMT constitutes an early process of tumor migration, we investigated whether TGF‐β/A549 cells could affect the EMT process. As shown in Figure 1F,G, western blot revealed that TGF‐β/A549 showed decreased expression of the epithelial maker E‐cadherin and increased expression of the mesenchymal makers N‐cadherin and vimentin, with immunofluorescence staining subsequently verifying this result (Figure 1H).

### 3.2 TGF‐β pretreated A549 cells increase vascular endothelial cell permeability and downregulate its tight junctions

**Figure 2:**
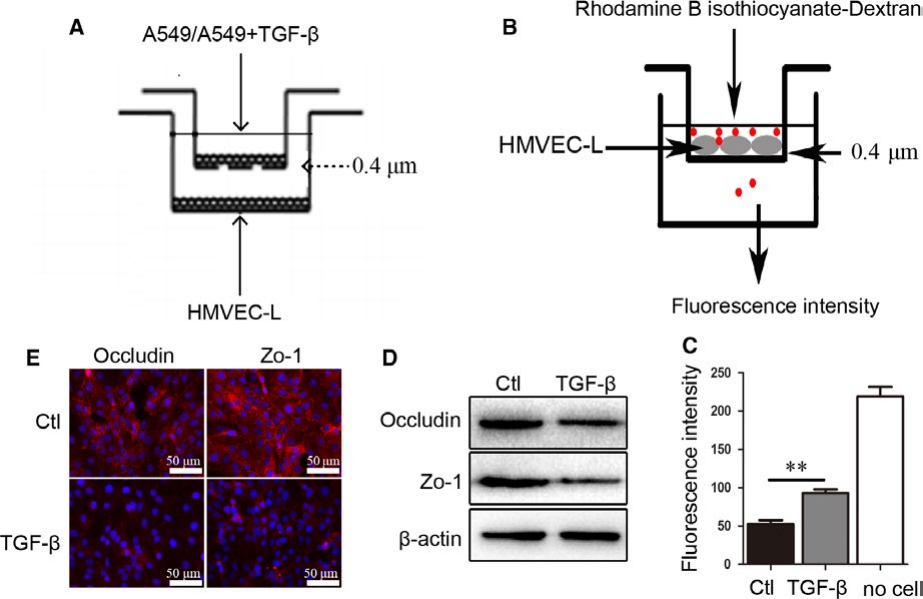
TGF‐β pretreated A549 cells increase HMVEC‐L monolayers permeability and downregulate tight‐junction protein expression. A, Schematic representation of the co‐culture assay. B, Schematic representation of the transwell chamber used for assaying transport across an endothelial monolayer. C, The permeability of pre‐cocultured HMVEC‐L monolayers grown on 0.4‐μm filters as measured by the appearance of rhodamine B isothiocyanate‐dextran, which was added in the upper well at the beginning of the experiment and in the bottom well after a 1‐h incubation. D and E, Expression of occludin and zonula occludens‐1 in A549/HMVEC‐L(Ctl) and TGF‐β+A549/HMVEC‐L cells (TGF‐β) as measured by western blot(D) and immunofluorescence staining(E) (scale bar, 50 μm). **P < 0.01 versus Ctl

We then cocultured HMVEC‐L cells with TGF‐β‐pretreated A549 cells (TGF‐β+A549) or untreated A549 cells (A549) and we designated as “TGF‐β” and “Ctl,” respectively (Figure 2A). We further evaluated vascular endothelial cell permeability according to rhodamine B isothiocyanate‐dextran penetration (Figure 2B). The optical density was measured in the lower wells to quantitatively assess rhodamine B isothiocyanate‐dextran transition through the vascular endothelial cell layer. We found that TGF‐β+A549/HMVEC‐L cells (TGF‐β) showed increased vascular endothelial cell permeability (Figure 2C), with western blot and immunofluorescence staining also revealing decreases in the expression of tight‐junction proteins in TGF‐β+A549/HMVEC‐L cells (TGF‐β) (Figure 2D,E).

### 3.3 TGF‐β‐mediated exosome release regulates lung cancer invasion and vascular permeability

**Figure 3:**
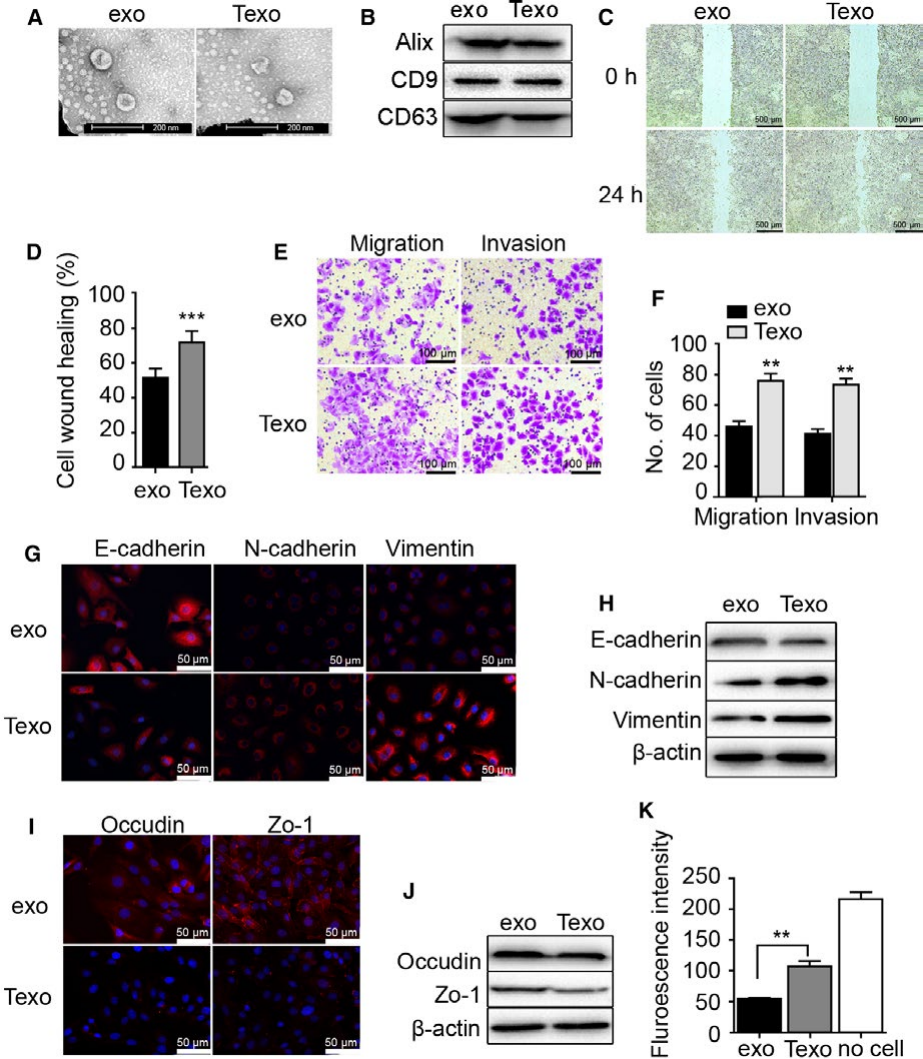
TGF‐β‐mediated exosome release promotes the migration and invasion of A549 cells and attenuates barrier functions at the HMVEC‐L monolayer. A, TEM images of exosomes secreted by A549 cells(exo) and TGF‐β‐pretreated A549 cells(Texo). B, exosome characteristic proteins Alix, CD9, CD63 were detected by western blot. C, Wound healing in exo and Texo treated A549 cells. Scale bar, 500 μm. D, Quantification of wound healing. E, Transwell migration and matrigel invasion by exo and Texo treated A549 cells. Scale bar, 100 μm. F, Quantification of transwell migration and matrigel invasion. G and H, Expression of E‐cadherin, N‐cadherin, and vimentin in exo and Texo treated A549 cells as measured by (G) immunofluorescence staining (scale bar, 50 μm) and (H) western blot. I and J, Expression of occludin and zonula occludens‐1 in exo and Texo treated HMVEC‐L cells as measured by (I) immunofluorescence staining (scale bar, 50 μm) and (J) western blot. K, Permeability of the exo and Texo treated HMVEC‐L monolayers. **P < 0.01; ***P < 0.001 versus exo

Recent studies showed that exosomes play important roles as carriers of intercellular signals during cancer invasion and vascular remodeling.^23^, ^24^ In the present study, we extracted exosomes from TGF‐β pretreated A549 cell culture supernatant (Texo) and untreated A549 cell culture supernatant (exo). To ensure successful isolation of exosomes, the collected exosomes were observed by transmission electron microscope (TEM; Figure 3A) and the exosome characteristic proteins Alix, CD9, CD63 were detected by western blot (Figure 3B). In our previous result, we have suggested that TGF‐β pretreated A549 cells regulated the function of A549 and HMVEC‐L cells. Here, to study whether the exosome contributes to the function changes, a series of assays were performed. With wound healing, transwell migration, and matrigel invasion assays revealing increased migration and invasiveness of Texo treated A549 cells relative to exo treated A549 cells (Figure 3C‐F). Immunofluorescence staining and western blot analysis revealed that Texo exhibited increased vimentin and N‐cadherin expression and decreased E‐cadherin expression in A549 cells, as well as decreased tight‐junction protein between vascular endothelial cells, which resulted in increased vascular endothelial cell permeability (Figure 3G‐K).

### 3.4 Lnc‐MMP2‐2 is highly enriched in TGF‐β‐mediated exosomes and alters MMP2 levels

**Figure 4:**
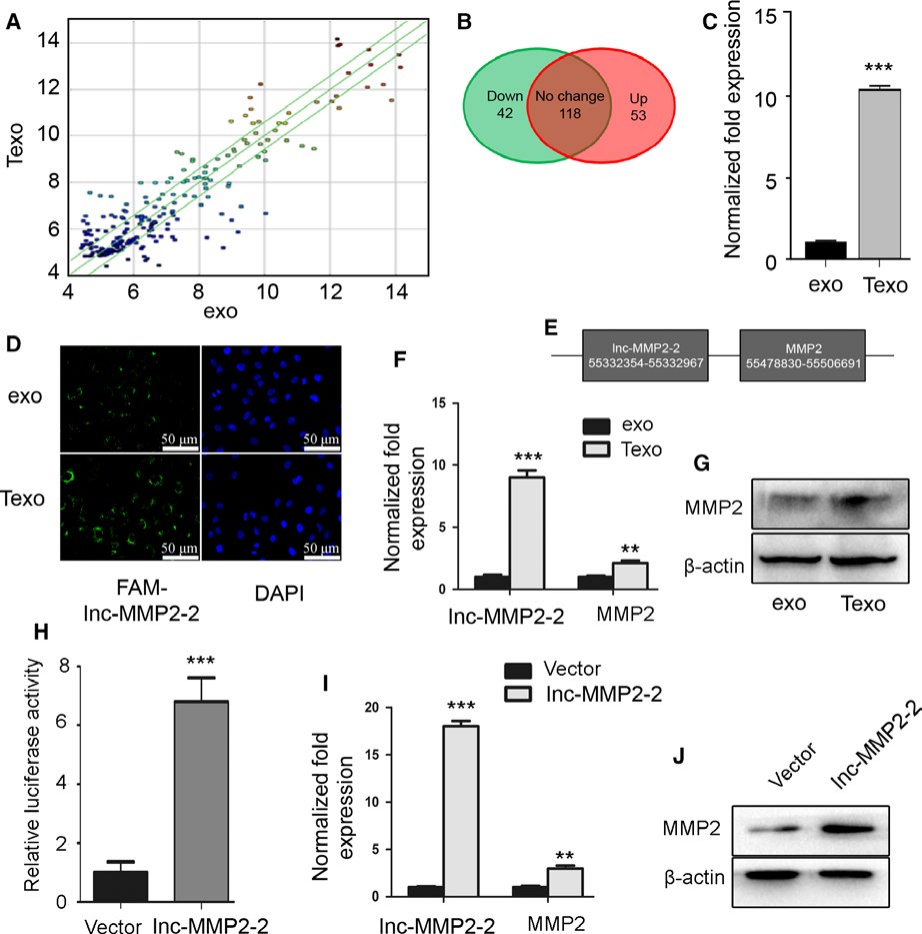
Lnc‐MMP2‐2 is highly enriched in TGF‐β‐mediated exosomes and alters MMP2 levels. A, Scatter Plots revealed differences in lncRNAs. B, Venn diagram revealed differences in lncRNAs. C, qRT‐PCR analysis of lnc‐MMP2‐2 levels in TGF‐β mediated A549 cell‐derived exosome (Texo) and Ctl exo(exo). D, FISH analysis of lnc‐MMP2‐2 levels in exosomes treated A549 cells. E, Gene location analysis shows that lnc‐MMP2‐2 is located in the upstream of MMP2, indicating its transcriptional enhancement potentiality. F and G, qRT‐PCR (F) and Western blot (G) analysis of the effects of the treatment of Texo in A549 cells on lnc‐MMP2‐2 and MMP2 expression. (G) Luciferase asssay was performed to evaluate the activity of the MMP2 promoter in A549 cells with lnc‐MMP2‐2 overexpression. I and J, qRT‐PCR (I) and Western blot (J) analyses of the effects of lnc‐MMP2‐2 overexpression on MMP2 expression. **P < 0.01; ***P < 0.001 versus exo

Recent studies showed that exosomal lncRNAs play a central role in cancer occurrence and metastasis.^25^ Using lncRNA microarray analysis, we screened for differentially expressed exosomal lncRNAs related to TGF‐β‐mediated EMT (Figure 4A,B). The top 20 over/underexpressed lncRNAs data is presented in Table S1. Bioinformatics analysis revealed possible regulatory mechanisms (Table 1) and specifically with those associated with maximal fold changes in lnc‐MMP2‐2. We next confirmed the high levels of lnc‐MMP2‐2 in TGF‐β‐mediated exosomes by qRT‐PCR (Figure 4C) and exosome treated A549 cells by FISH (Figure 4D). Our results also suggested a possible role in increasing MMP2 levels, with gene location analysis indicating the presence of a lnc‐MMP2‐2‐binding site located upstream of the MMP2 gene and suggesting a potential role as a transcriptional enhancer (Figure 4E). Subsequently, with qRT‐PCR and western blot assay, we observed that lnc‐MMP2‐2 and MMP2 were both up‐regulated in Texo treated A549 cells (Figure 4F,G). Additionally, to investigate the relationship between lnc‐MMP2‐2 and MMP2, MMP2 promoter‐Luciferase asssay, qRT‐PCR and western blot assays were performed. With Luciferase asssay, we observed that the relative luciferase activity was markedly increased when MMP2 promoter was co‐transfected with lnc‐MMP2‐2 in A549 cells (Figure 4H). To further study whether lnc‐MMP2‐2 can directly increased the expression of MMP2, we overexpressed lnc‐MMP2‐2 in A549 cells and qRT‐PCR (Figure 4I) and western blot (Figure 4J) confirmed our hypothesis.

**Table 1:** Differential lncRNAs and bioinformatics analysis of the top 20 possible regulatory mechanisms

LncRNA information				Relationship		Potential target‐gene information	
Gene Symbol	Fold change	RNA length	Coordinates	Potential Mechanism	Binding miRNA	Symbol	Coordinates
lnc‐MMP2‐2	11.614376	613	chr16:55366266‐55366879(+)	Enhancer^a^		MMP2	chr16:55513080‐55540586(+)
lnc‐MMP2‐2	11.614376	613	chr16:55366266‐55366879(+)	ceRNA^b^	hsa‐miR‐30c‐2‐3p,hsa‐miR‐185‐5p	EPB41L5	chr2:120770603‐120936697(+)
lnc‐MMP2‐2	11.614376	613	chr16:55366266‐55366879(+)	ceRNA	hsa‐miR‐30c‐2‐3p,hsa‐miR‐30c‐1‐3p	MT1F	chr16:56691854‐56693215(+)
lnc‐MMP2‐2	11.614376	613	chr16:55366266‐55366879(+)	ceRNA	hsa‐miR‐637,hsa‐miR‐3192	WNT5A	chr3:55499742‐55521670(−)
lnc‐MMP2‐2	11.614376	613	chr16:55366266‐55366879(+)	ceRNA	hsa‐miR‐625‐5p,hsa‐miR‐940	ETS1	chr11:128328655‐128392205(−)
lnc‐MMP2‐2	11.614376	613	chr16:55366266‐55366879(+)	ceRNA	hsa‐miR‐24‐3p,hsa‐miR‐30c‐2‐3p	MT1E	chr16:56659584‐56661024(+)
lnc‐MMP2‐2	11.614376	613	chr16:55366266‐55366879(+)	ceRNA	hsa‐miR‐30c‐2‐3p,hsa‐miR‐30c‐1‐3p	BMI1	chr10:22610138‐22620414(+)
lnc‐MMP2‐2	11.614376	613	chr16:55366266‐55366879(+)	ceRNA	hsa‐miR‐24‐3p,hsa‐miR‐30c‐2‐3p	MT1M	chr16:56666144‐56667898(+)
RP11‐89H19.1	−10.37806	1287	chr12:48276431‐48295308(+)	ceRNA	hsa‐miR‐204‐3p,hsa‐miR‐214‐3p	MCM5	chr22:35796115‐35820495(+)
AC005592.2	−10.02904	566	chr5:141783765‐142051566(+)	Neighboring^c^		FGF1	chr5:141971742‐142066060(−)
RP11‐390F4.10	−6.100435	422	chr9:6704470‐6707780(+)	Enhancer		KDM4C	chr9:6757640‐7175648(+)
RP3‐419C19.3	5.0700395	378	chr1:192765662‐192766335(+)	Enhancer		RGS2	chr1:192778168‐192781407(+)
XLOC_007882	−5.030651	1570	chr9:136999206‐137001037(−)	ceRNA	hsa‐miR‐185‐3p,hsa‐miR‐762	FOXC2	chr16:86600856‐86602537(+)
XLOC_007882	−5.030651	1570	chr9:136999206‐137001037(−)	Neighboring		WDR5	chr9:137001209‐137025094(+)
XLOC_007882	−5.030651	1570	chr9:136999206‐137001037(−)	ceRNA	hsa‐miR‐608,hsa‐miR‐762	EGFR	chr7:55086724‐55275031(+)
XLOC_007882	−5.030651	1570	chr9:136999206‐137001037(−)	ceRNA	hsa‐miR‐762,hsa‐miR‐1225‐3p	PTBP1	chr19:797391‐812327(+)
XLOC_007882	−5.030651	1570	chr9:136999206‐137001037(−)	ceRNA	hsa‐miR‐762,hsa‐miR‐286	FLNB	chr3:57994126‐58157982(+)
XLOC_007882	−5.030651	1570	chr9:136999206‐137001037(−)	ceRNA	hsa‐miR‐34a‐5p,hsa‐miR‐185‐3p	JAG1	chr20:10618331‐10654694(−)
XLOC_007882	−5.030651	1570	chr9:136999206‐137001037(−)	Neighboring		WDR5	chr9:137001209‐137025094(+)
XLOC_014378	−5.02524	1555	chr22:39608718‐39610817(−)	Enhancer		PDGFB	chr22:39619684‐39640957(−)

a Enhancer: lncRNA‐binding sites located within 300 kb of the potential target genes and that might regulate gene expression at the transcription level.

b ceRNA: lncRNAs that are potential ceRNAs of genes and that share miRNA‐response elements with mRNA transcripts of the corresponding target genes.

c Neighboring: lncRNAs that overlap in part with critical pathway genes or with binding sites located within 3 kb from genes and that might regulate neighboring genes at the transcription or posttranscription level.

For TGF‐β may be coisolated with the exosome preparation and cause the effects observed, we especially detected the soluble TGF‐β in exo and Texo, As expected, we did not detected TGF‐β out in both exo and Texo (Figure S1).

### 3.5 Ectopic expression or silencing of lnc‐MMP2‐2 mediates lung cancer invasion and vascular permeability

**Figure 5:**
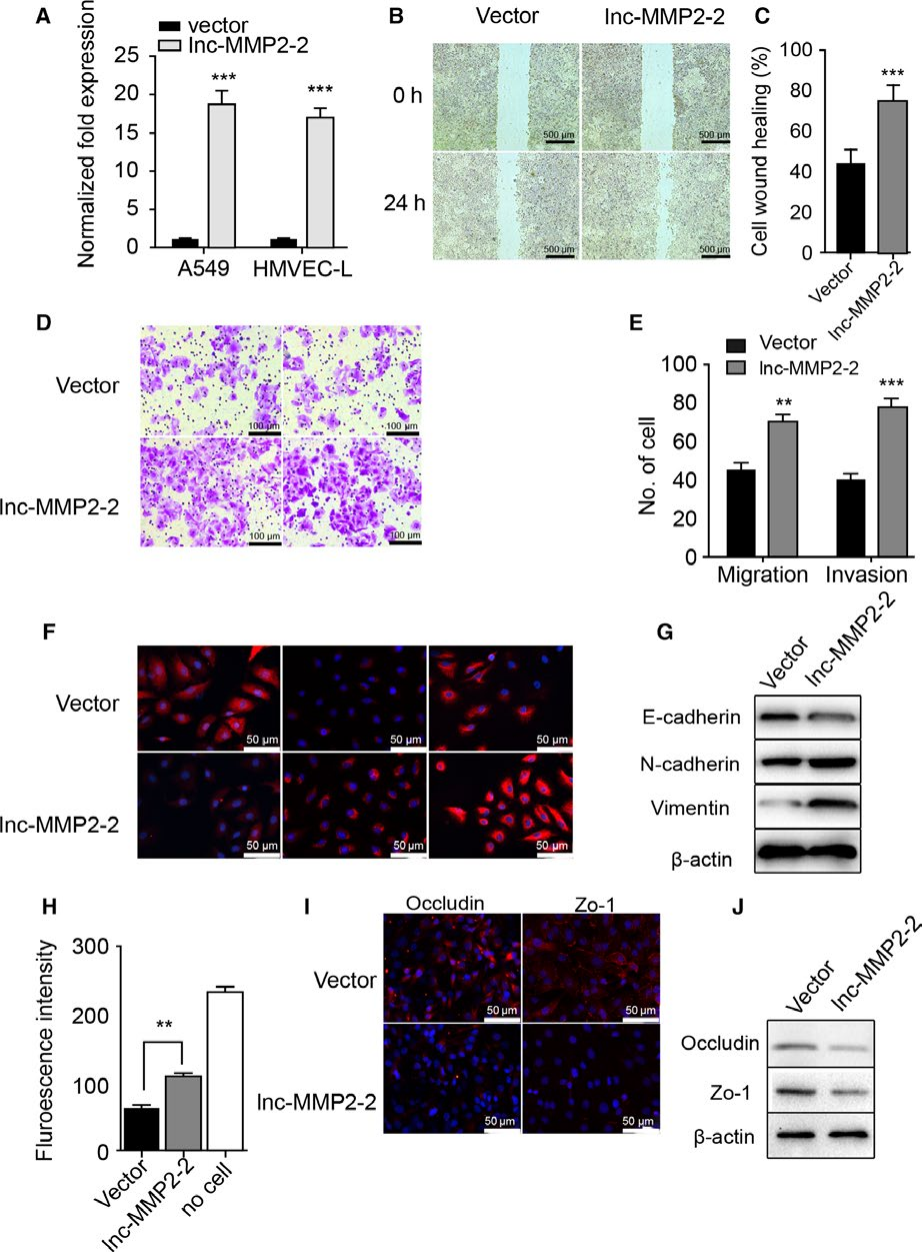
Overexpression of lnc‐MMP2‐2 promotes migration and invasion of A549 cells and attenuates barrier functions at the HMVEC‐L monolayer. A, Lnc‐MMP2‐2 expression in control (Vector) and lnc‐MMP2‐2‐transfected (lnc‐MMP2‐2) A549 and HMVEC‐L cells as measured by qRT‐PCR. B, Wound healing in Vector and lnc‐MMP2‐2 A549 cells. Scale bar, 500 μm. C, Quantification of wound healing. D, Transwell migration and matrigel invasion by Vector and lnc‐MMP2‐2 A549 cells. Scale bar, 100 μm. E, Quantification of transwell migration and matrigel invasion. F and G, Expression of E‐cadherin, N‐cadherin, and vimentin in Vector and lnc‐MMP2‐2 A549 cells as measured by (F) immunofluorescence staining (scale bar, 50 μm) and (G) western blot. H and I, Expression of occludin and zonula occludens‐1 in Vector and lnc‐MMP2‐2 HMVEC‐L cells as measured by (H) immunofluorescence staining (scale bar, 50 μm) and (I) western blot. J, Permeability of Vector and lnc‐MMP2‐2 HMVEC‐L monolayers. **P < 0.01; ***P < 0.001 versus Vector

Ectopic expression was confirmed following transfection of pCDNA‐3.1‐lnc‐MMP2‐2 in A549 cells and HMVEC‐L, respectively (Figure 5A). We then verified that lnc‐MMP2‐2 overexpression increased A549‐cell migration and invasion (Figure 5B‐E), and immunofluorescence staining and western blot confirmed increases in vimentin and N‐cadherin expression and decreases in E‐cadherin expression, as well as that of tight‐junction proteins between vascular endothelial cells (Figure 5F,G,I, J), and increased permeability of HMVEC (Figure 5H).

**Figure 6:**
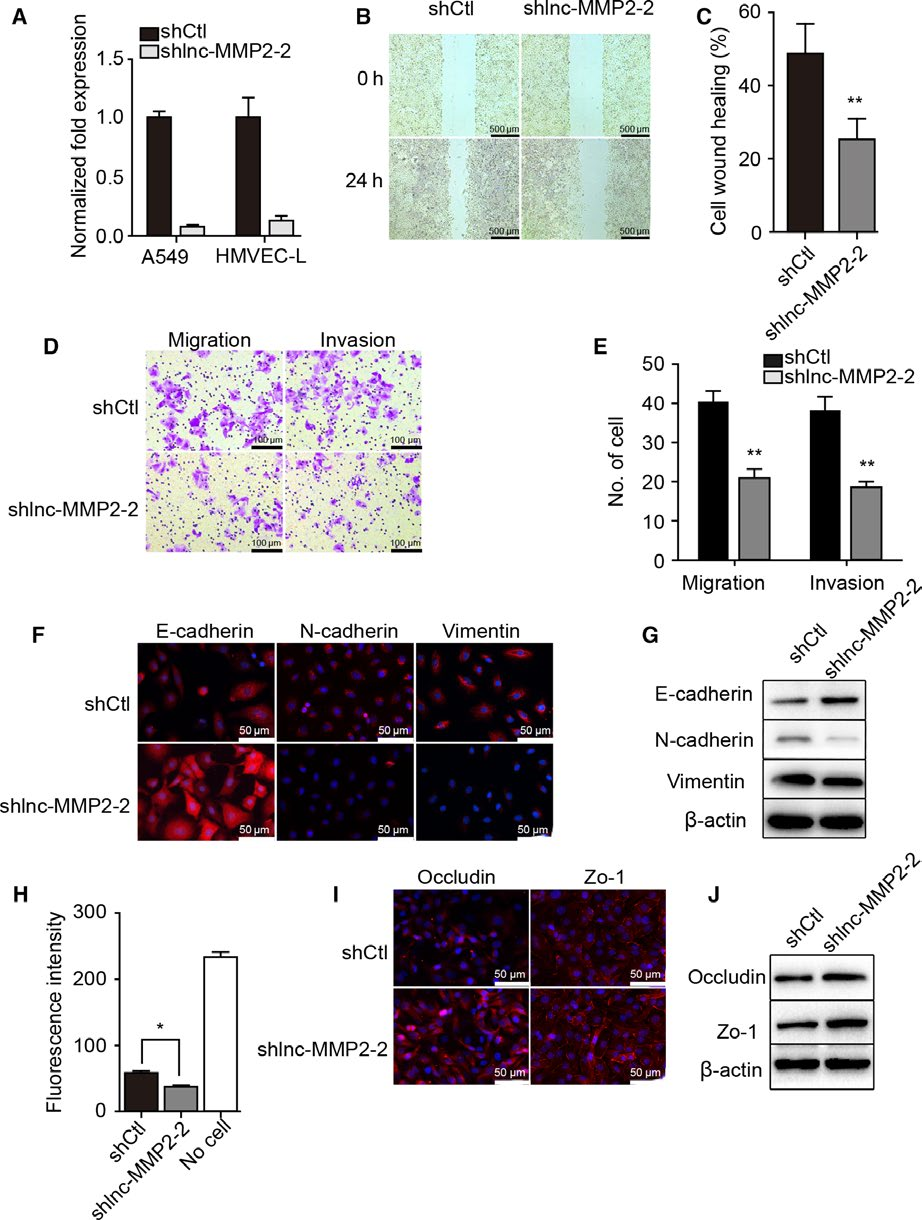
Lnc‐MMP2‐2 silencing inhibits the migration and invasion of A549 cells and protects barrier functions at the HMVEC‐L monolayer. A, Lnc‐MMP2‐2 expression in control (shCtl) and knockdown (shlnc‐MMP2‐2) A549 and HMVEC‐L cells as measured by qRT‐PCR. B, Wound healing by shCtl and shlnc‐MMP2‐2 A549 cells. Scale bar, 500 μm. C, Quantification of wound healing. D, Transwell migration and matrigel invasion by shCtl and shlnc‐MMP2‐2 A549 cells. Scale bar, 100 μm. E, Quantification of transwell migration and matrigel invasion. F and G, Expression of E‐cadherin, N‐cadherin, and vimentin in shCtl and shlnc‐MMP2‐2 A549 cells as measured by (F) immunofluorescence staining (scale bar, 50 μm) and (G) western blot. H and I, Expression of occludin and zonula occludens‐1 in shCtl and shlnc‐MMP2‐2 HMVEC‐L cells as measured by (H) immunofluorescence staining (scale bar, 50 μm) and (I) western blot. J, Permeability of shCtl and shlnc‐MMP2‐2 HMVEC‐L monolayers. *P < 0.05; **P < 0.01 versus shCtl

To evaluate alterations following lnc‐MMP2‐2 knockdown, we silenced lnc‐MMP2‐2 expression (Figure 6A), and evaluated the metastatic potential of lung cancer cells and permeability of vascular endothelial cell following depletion of lnc‐MMP2‐2. Our results revealed that lnc‐MMP2‐2 silencing inhibited A549‐cell migration and invasion (Figure 6B‐E), reduced vimentin and N‐cadherin expression, increased E‐cadherin expression, as well as that of tight‐junction proteins between vascular endothelial cells (Figure 6F,G,I,J), and decreased permeability of HMVEC (Figure 6H).

### 3.6 Lnc‐MMP2‐2 expression is positively correlated with MMP2 expression

**Figure 7:**
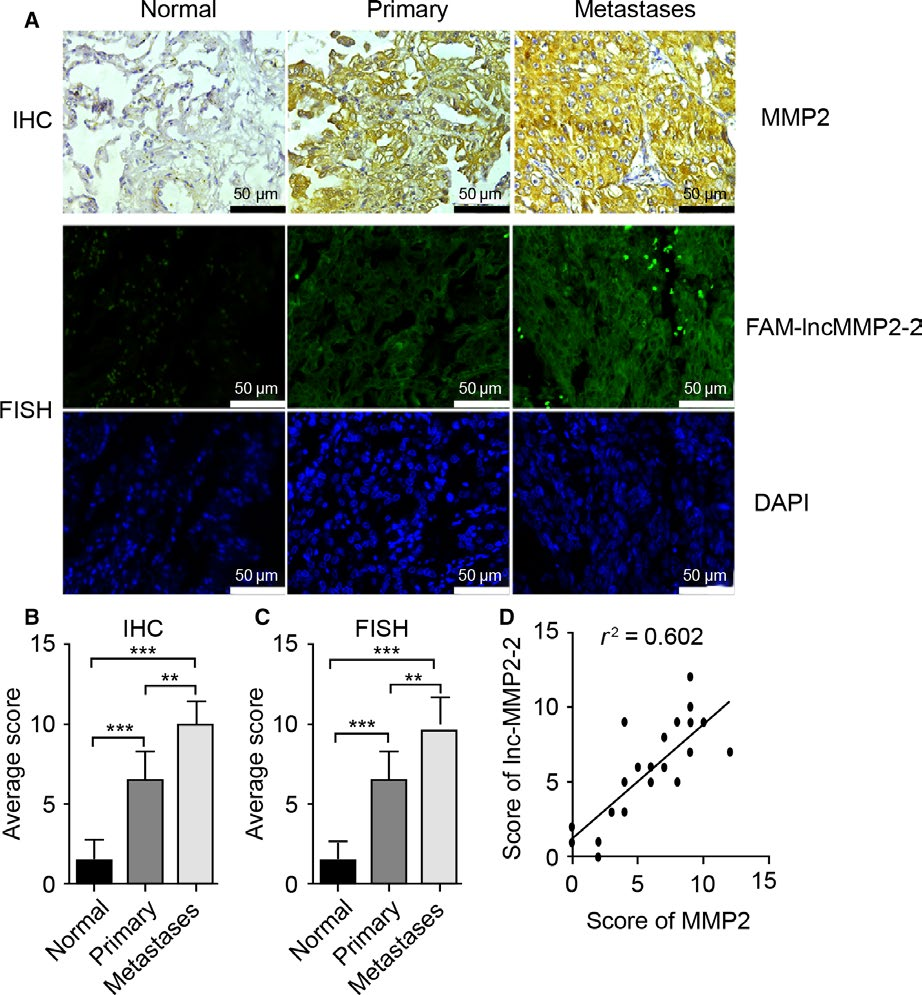
Lnc‐MMP2‐2 is associated with MMP2 expression and metastatic progression in lung cancer. A, Representative images of MMP2 and lnc‐MMP2‐2 staining in normal, primary, and metastatic lung tissue. Scale bar, 50 μm. B, Immunohistochemistry scores for MMP2 in normal (n* *=* *5), primary (n* *=* *14), and metastatic (n* *=* *4) lung tissue. C, Fluorescence in situ hybridization scores for lnc‐MMP2‐2 in normal (n* *=* *5), primary (n* *=* *14), and metastatic (n* *=* *4) lung tissue. D, Correlation between MMP2 and lnc‐MMP2‐2 in all cases (n* *=* *23). **P < 0.01; ***P < 0.001

Lung cancer tissue microarray analysis showed gradual and correlated increases in MMP2 and lnc‐MMP2‐2 expression associated with lung cancer progression from normal lung tissue to primary lung cancer tissue to metastatic lung cancer (Figure 7A). Additionally, semiquantitative analysis of MMP2 and lnc‐MMP2‐2 expression confirmed positive correlations between MMP2 and lnc‐MMP2‐2 levels during lung cancer progression (Figure 7B‐D).

## 4 DISCUSSION

The EMT plays a significant role during tumor invasion and metastasis and is characterized by the acquisition of mesenchymal markers and loss of epithelial‐cell‐adhesion molecules.^26^ To investigate whether pretreatment with TGF‐β, an inducer of EMT, affects the metastatic potential of lung cancer cells, we constructed a coculture model. We found that coculture of cells with TGF‐β‐pretreated cells increased the metastatic potential of the cocultured lung cancer cells, as well as vascular endothelial cell permeability. This result indicated that the high metastatic potential of lung cancer cells can affect other lung cancer cells and regulate vascular permeability by secreting or releasing certain substances.

Exosomes carry proteins and genetic material and play important roles in signal transduction between cells.^21^ In the present study, we found that exosomes extracted from the cell culture supernatant TGF‐β‐pretreated A549 cells were capable of increasing the metastatic potential of lung cancer cells and enhancing vascular endothelial cell permeability, indicating that exosomes derived from lung cancer cells with high metastatic potential could affect other lung cancer cells and regulate vascular permeability.

Recent studies showed that exosomal lncRNAs play an important role in signal exchange between tumor cells, the local microenvironment, or distal target organs.^15^ In the present study, analysis of a lncRNA lung cancer tissue microarrays revealed differences in exosomal lncRNA expression based on cancer status and identified lnc‐MMP2‐2 as exhibiting high degrees of differential expression during this process. Bioinformatics analysis identified lnc‐MMP2‐2 as an “enhancer‐like LncRNA,” with potential binding sites located within 300 kb of possible target genes to function as a positive regulator of expression at the transcriptional level.^15^ Subsequent analysis revealed a lnc‐MMP2‐2‐binding site located upstream of MMP2.

Previous studies reported roles for MMP2 in regulating lung cancer invasion and vascular permeability.^27^, ^28^, ^29^ In the present study, exogenous overexpression and interference experiments showed that lnc‐MMP2‐2 promoted lung cancer invasion and increased vascular permeability, and that lnc‐MMP2‐2 expression was markedly higher in lung cancer tissues than in normal controls. Furthermore, we found that lnc‐MMP2‐2 levels were positively correlated with MMP2 levels during lung cancer progression. In conclusion, we demonstrated that lnc‐MMP2‐2 was overexpressed in lung cancer tissue and correlated with increased migration and invasion. Moreover, exosomal lnc‐MMP2‐2 promoted MMP2 expression in TGF‐β‐mediated lung cancer invasion and increased vascular permeability. Thus, these findings suggested that exosomal lnc‐MMP2‐2 might represent a prognostic biomarker of increased metastatic stage and a putative therapeutic target for lung cancer treatment. Future work is necessary to determine the extent of the role of lnc‐MMP2‐2 in lung cancer pathophysiology.

## CONFLICT OF INTEREST

The authors declare no competing financial interests.
