# Corrigendum

**DOI:** 10.1002/cam4.3145

**Published:** 2020-06-14

In the article by Wu et al,^1^ the authors have noticed that the lnc‐MMP2‐2 primer sequences listed in section 2.6 “Total RNA extraction and quantitative reverse transcription polymerase chain reaction (qRT‐PCR)” are incorrect. To use the same forward and reverse primers in PCR is almost impossible.

The correct forward and reverse lnc‐MMP2‐2 primer sequences should be: TCCATCCTGCTGCTCAGTATCTCC and GCTCAGACGTGCCATTCTCAGG.

The authors apologize for this error.
