# Supplementary figures and images for: TGF‐β‐mediated exosomal lnc‐MMP2‐2 regulates migration and invasion of lung cancer cells to the vasculature by promoting MMP2 expression
Source: Cancer Med. 2018 Sep 6;7(10):5118–29. doi: 10.1002/cam4.1758 (PMC6198203; doi:10.1002/cam4.1758)

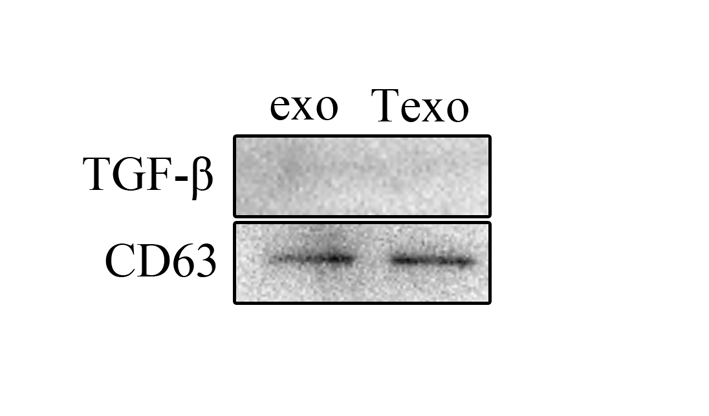

Supplement: Supplementary file 1 [file CAM4-7-5118-s001.tif]
