# Supplementary material for: TGF‐β‐mediated exosomal lnc‐MMP2‐2 regulates migration and invasion of lung cancer cells to the vasculature by promoting MMP2 expression
Source: Cancer Med. 2018 Sep 6;7(10):5118–29. doi: 10.1002/cam4.1758 (PMC6198203; doi:10.1002/cam4.1758)
Supplement: Supplementary file 2 [file CAM4-7-5118-s002.docx]

**Supplementary Table 1: The top 20 over/under-expressed lncRNAs between exosomes derived form untreated (exo) or TGF-β pretreatedA549 cell culture supernatant (Texo).**

| **LncRNA information** | | | | | |  | | **Raw Intensity** | | **Fold Change** | **Regulation** |
| --- | --- | --- | --- | --- | --- | --- | --- | --- | --- | --- | --- |
| **GeneName** | **chrom** | **strand** | **txStart** | **txEnd** | **RNA length** |  | | **[Texo]**  **(raw)** | **[exo]**  **(raw)** |  |  |
| Lnc-MMP2-2 | chr16 | + | 55366266 | 55366879 | 613 |  | | 303 | 45.7 | 11.6 | up |
| AC097359.2 | chr3 | + | 37237694 | 37238157 | 463 |  | | 250 | 26.1 | 7.43 | up |
| XLOC_008474 | chr10 | + | 45073474 | 45086387 | 372 |  | | 229 | 29.9 | 5.44 | up |
| RP3-419C19.3 | chr1 | + | 192765662 | 192766335 | 378 |  | | 6483 | 566 | 5.07 | up |
| MTND4P26 | chr2 | + | 120971206 | 120972565 | 1359 |  | | 224 | 35.5 | 4.24 | up |
| LINC00528 | chr22 | + | 18260055 | 18262247 | 2192 |  | | 4644 | 478 | 4.24 | up |
| AC104438.1 | chr3 | + | 65573176 | 65573725 | 545 |  | 118 | | 25.5 | 4.06 | up |
| CYP4A22-AS1 | chr1 | - | 47562324 | 47644943 | 921 |  | 871 | | 116 | 3.92 | up |
| BC035889 | chr7 | + | 27161537 | 27168379 | 2020 |  | 14 | | 3097 | 3.82 | up |
| RP11-986E7.2 | chr14 | - | 95141634 | 95142166 | 532 |  | 104 | | 24.7 | 3.71 | up |
| CECR3 | chr22 | - | 17737749 | 17747623 | 1915 |  | 331 | | 59.9 | 3.4 | up |
| HYMAI | chr6 | - | 144324033 | 144329867 | 5005 |  | 824 | | 124 | 3.38 | up |
| XLOC_002674 | chr3 | + | 58165850 | 58173195 | 751 |  | 2060 | | 337 | 3.14 | up |
| RP11-619A14.2 | chr11 | + | 75901768 | 75906115 | 613 |  | 97.6 | | 25.4 | 3.12 | up |
| CDKN2B-AS1 | chr9 | + | 21994789 | 22121093 | 1805 |  | 22203 | | 3144 | 3.12 | up |
| RP3-512B11.3 | chr6 | - | 7540683 | 7541571 | 888 |  | 14 | | 3318 | 3.1 | up |
| PPIAP27 | chr13 | - | 21530317 | 21530582 | 265 |  | 3144 | | 485 | 2.96 | up |
| XLOC_007277 | chr9 | + | 7208935 | 7209962 | 206 |  | 117 | | 31.8 | 2.75 | up |
| CTD-2561B21.10 | chr17 | - | 78997344 | 78999303 | 368 |  | 2479 | | 427 | 2.74 | up |
| RP4-607I7.1 | chr11 | - | 35154201 | 35159579 | 1102 |  | 75.9 | | 24.1 | 2.6 | up |
| RP11-89H19.1 | chr12 | + | 48276431 | 48295308 | 1287 |  | 129 | | 634 | -10 | down |
| AC005592.2 | chr5 | + | 141783765 | 142051566 | 566 |  | 74.7 | | 371 | -10 | down |
| RP11-390F4.10 | chr9 | + | 6704470 | 6707780 | 422 |  | 106 | | 339 | -6.1 | down |
| XLOC_000587 | chr1 | + | 225898089 | 225904316 | 750 |  | 62.9 | | 226 | -6.1 | down |
| CTD-2561B21.11 | chr17 | - | 78991768 | 78993811 | 869 |  | 4301 | | 7961 | -5.1 | down |
| XLOC_007882 | chr9 | - | 136999206 | 137001037 | 1570 |  | 162 | | 393 | -5 | down |
| XLOC_014378 | chr22 | - | 39608718 | 39610817 | 1555 |  | 26.7 | | 86.5 | -5 | down |
| CBR3-AS1 | chr21 | - | 37504064 | 37528606 | 1500 |  | 975 | | 1639 | -4.2 | down |
| RP3-340I3.1 | chr12 | - | 1609690 | 1616484 | 2417 |  | 126 | | 294 | -4.1 | down |
| RP11-293M10.4 | chr14 | - | 75758829 | 75759198 | 369 |  | 59.5 | | 108 | -3.3 | down |
| AP000439.2 | chr11 | - | 69253132 | 69259511 | 581 |  | 30.6 | | 65.2 | -3.3 | down |
| RP11-429J17.8 | chr8 | + | 144871684 | 144872445 | 428 |  | 31.7 | | 67.5 | -3.2 | down |
| RP11-192I3.2 | chr1 | - | 228162979 | 228163386 | 399 |  | 1052 | | 1442 | -3 | down |
| RP4-543J13.1 | chr1 | + | 114749048 | 114804900 | 332 |  | 65.5 | | 105 | -2.9 | down |
| uc.338 | chr12 | + | 53858488 | 53858711 | 223 |  | 35 | | 64 | -2.8 | down |
| XLOC_013785 | chr20 | - | 48642151 | 48647008 | 499 |  | 6425 | | 6099 | -2.7 | down |
| RP11-823P9.3 | chr5 | - | 21481435 | 21484120 | 2685 |  | 10842 | | 11398 | -2.3 | down |
| MEG3 | chr14 | + | 101292468 | 101327360 | 1731 |  | 55.9 | | 81.4 | -2.3 | down |
| AC006159.4 | chr7 | - | 116203647 | 116211288 | 692 |  | 66 | | 88.7 | -2.2 | down |
| RP3-481F12.1 | chr20 | + | 55903601 | 55903752 | 151 |  | 31.4 | | 46 | -2.2 | down |
